# Rapid Hydrophilization of Model Polyurethane/Urea (PURPEG) Polymer Scaffolds Using Oxygen Plasma Treatment

**DOI:** 10.3390/polym8040144

**Published:** 2016-04-15

**Authors:** Rok Zaplotnik, Alenka Vesel, Gregor Primc, Xiangyu Liu, Kevin C. Chen, Chiju Wei, Kaitian Xu, Miran Mozetic

**Affiliations:** 1 Jozef Stefan Institute, Jamova cesta 39, 1000 Ljubljana, Slovenia; alenka.vesel@ijs.si (A.V.); gregor.primc@ijs.si (G.P.); miran.mozetic@guest.arnes.si (M.M.); 2 Multidisciplinary Research Center, Shantou University, Shantou 515063, China; 13xyliu3@stu.edu.cn (X.L.); ckchen@stu.edu.cn (K.C.C.); chijuwei@stu.edu.cn (C.W.); kaitianxu@yahoo.com (K.X.)

**Keywords:** polyurea, polyurethane, oxygen plasma treatment, RF power, surface modification, surface reaction mechanisms, optical emission spectroscopy, residual gas analysis, mass spectrometry

## Abstract

Polyurethane/urea copolymers based on poly(ethylene glycol) (PURPEG) were exposed to weakly ionized, highly reactive low-pressure oxygen plasma to improve their sorption kinetics. The plasma was sustained with an inductively coupled radiofrequency generator operating at various power levels in either E-mode (up to the forward power of 300 W) or H-mode (above 500 W). The treatments that used H-mode caused nearly instant thermal degradation of the polymer samples. The density of the charged particles in E-mode was on the order of 10^16^ m^−3^, which prevented material destruction upon plasma treatment, but the density of neutral O-atoms in the ground state was on the order of 10^21^ m^−3^. The evolution of plasma characteristics during sample treatment in E-mode was determined by optical emission spectroscopy; surface modifications were determined by water adsorption kinetics and X-ray photoelectron spectroscopy; and etching intensity was determined by residual gas analysis. The results showed moderate surface functionalization with hydroxyl and carboxyl/ester groups, weak etching at a rate of several nm/s, rather slow activation down to a water contact angle of 30° and an ability to rapidly absorb water.

## 1. Introduction

Tissue engineering is a rapidly expanding multidisciplinary scientific field with promising applications in biomedicine. Non-cancerous cells do not grow three-dimensionally; therefore, they need appropriate scaffolds to form three-dimensional tissues. The ideal scaffolds should allow the seeded cells to adhere and proliferate, enable the mobility of nutrients, and be made from biodegradable materials. Commonly used materials that satisfy the above requirements include polylactic acid (PLA), polyglycolic acid (PGA) and polycaprolactone (PCL) [1,2]. Scaffolds made from these materials often give satisfactory results; however, researchers worldwide are still seeking other materials suitable for particular applications. Polyurea copolymers may have optimal biocompatibility and the appropriate physical and chemical properties. Block polyurethanes could be promising candidates for nerve repair scaffolds [3,4]. Recently published results on their *in vivo* and *in vitro* applications have demonstrated satisfactory nerve regeneration with better performance than using autografts [3,4]. Nevertheless, the surface properties of such materials and their ability to adsorb and distribute liquids may not be optimal.

Cell adhesion and proliferation on scaffolds depends on numerous parameters, including the surface energy of the material used. Several authors have addressed the adhesion and multiplication of different biological cells on materials as a function of the material’s composition and structure [5,6,7,8,9]. The reported results vary significantly; however, one robust conclusion is that the best results are obtained by using moderately hydrophilic substrates [10,11]. The majority of biocompatible polymers are moderately hydrophobic, often with water contact angles between 70° and 100°. PLA exhibits a contact angle in the range of 61° to 87° [12,13,14,15,16], with a typical value of approximately 80° [13,14]; for PGA, a contact angle of 53° was reported [17]; and for PCL fiber and PCL mesh, contact angles of 86° [18] and 130° [19,20] were reported, respectively.

The surface properties of porous and/or fibrous polymers suitable for synthesizing scaffolds for tissue engineering can be tailored with different treatments; however, one of the best techniques is the application of gaseous plasma [21]. Gaseous molecules partially ionize, dissociate and excite under plasma conditions [22,23], and the resulting reactive gaseous species interact chemically with the polymer surface to form new functional groups. The types of functional groups formed depends on the polymer’s properties, the type of gas and the plasma parameters. Polymers become more hydrophobic if they are functionalized with non-polar functional groups, usually by using fluorine-containing gases [24]. Hydrophilization, on the other hand, is achieved by functionalization with polar groups, which is usually achieved by treatment with plasma using oxygen-containing gases [25,26,27]. Because the required surface finish of materials used for scaffolds is moderately hydrophilic, oxygen plasma is often applied to polymeric scaffolds [5,6]. The plasma treatment may or may not lead to the preferred surface finish. Some authors report rapid functionalization as well as a change in surface wettability [28], whereas others obtain rather unsatisfactory results. Poor functionalization with polar groups (or absence of these groups) upon treatment of fluorinated polymers by weakly ionized oxygen plasma is explained by extensive etching [29,30]: reactive oxygen species do interact chemically with fluorinated polymer but the molecular fragments containing oxygen desorb from the surface immediately. The functionalization of some other types of polymers often does not lead to satisfactory surface functionalization upon treatment with reactive oxygen species due to preferential etching [31,32]. The discrepancies in reported results may arise because most authors do not report the details of treatment conditions, but rather represent plasma as a black box. Hydrophobic recovery, which is typical in oxygen plasma treated polymers, is frequently neglected.

Although numerous authors have addressed the modification of the surface properties of materials that are suitable for tissue engineering using gaseous plasma treatment, there have only been a handful of reports on the functionalization of polyurethane and polyethylene glycol. Furthermore, to our knowledge, there is only one published paper in which polyurethane/urea copolymers based on polyethylene glycol were modified with plasma treatment [33]. In that case, the polymer was treated with ammonia plasma; whereas in this paper, we report modifications of this type of polymer using oxygen plasma treatment while adjusting different treatment parameters.

## 2. Materials and Methods

### 2.1. Sample Preparation

**Figure 1:**
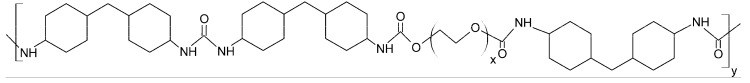
Chemical structure of Polyurethane/urea copolymers based on poly(ethylene glycol) (PURPEG) polymer.

**Figure 2:**
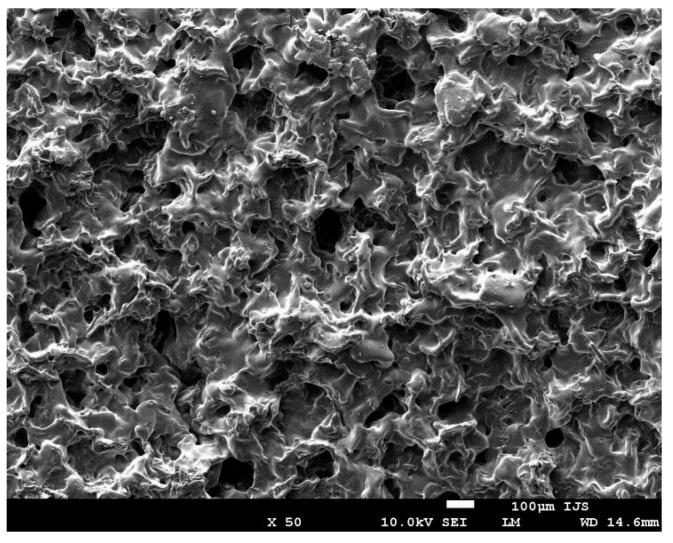
SEM image of an untreated PURPEG sample.

Polyurethane/urea copolymers (PUR) based on poly(ethylene glycol) (PEG), hereafter PURPEG, with the chemical structure presented in Figure 1, were prepared as a foam using a standard emulsification and freeze-drying procedure. The preparation procedure of the PURPEG polymer is explained in more detail in our recently published study [33]. As shown in the image in Figure 2, which was recorded by scanning electron microscopy (SEM), the samples were highly porous. The porosity was approximately 70% with a surface and interior pore size of 30–90 and 70–130 μm, respectively.

### 2.2. Plasma Treatment

**Figure 3:**
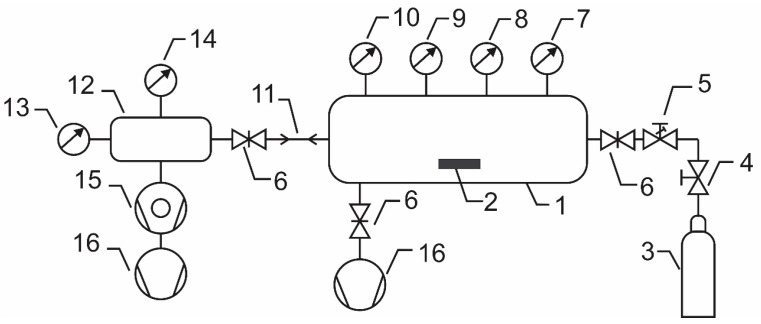
Schematic of the experimental setup: 1—plasma reactor, 2—sample, 3—oxygen flask, 4—high pressure valve, 5—leak valve, 6—gate valves, 7—double electrical probe, 8—optical spectrometer, 9—catalytic probe, 10—absolute vacuum gauge, 11—capillary, 12—high vacuum chamber, 13—high vacuum gauge, 14—residual gas analyzer, 15—turbomolecular pump, 16—rotary pumps.

The samples of PURPEG polymer were cut into small pieces with lateral dimensions of approximately 1 cm × 2 cm immediately before treatment with gaseous plasma. We used the plasma reactor represented schematically in Figure 3. The reactor had a volume of 1 liter and was separated from the vacuum pump by a gate valve. Once the sample was placed in the reactor, the reactor was pumped down to the ultimate pressure using a two-stage rotary pump, which was mounted to the plasma chamber via a gate valve. The reactor was equipped with an absolute vacuum gauge calibrated for a range of pressures between 1 and 1000 Pa. The ultimate pressure was typically 1 or 2 Pa; however, this value includes some uncertainty because it is at the limit of the gauge’s sensitivity. Once the plasma chamber was evacuated, the gate valve between the chamber and the rotary pump was closed, and the leak valve was carefully opened until the pressure increased to 33 Pa. The quantity of gas inside the reactor after closing the leak valve remained nearly constant over a period of several minutes. The number of gaseous molecules in the reactor was calculated from a known volume (1 liter) and pressure (33 Pa) using the relation: *N*_O2_ = *p*·*V*/*k*_B_·*T*, where *p* is the measured pressure, *V* is the reactor volume, *k*_B_ is the Boltzmann constant and *T* is the absolute temperature (approximately 300 K). Using the numerical values for the reactor, the calculated number of oxygen molecules was *N*_O2_ = 7.9 × 10^18^.

The plasma was sustained in a plasma reactor made from borosilicate glass using a radiofrequency (RF) generator, which operated at a frequency of 13.56 MHz and an adjustable forward power up to 1200 W. The generator was coupled inductively via a matching network. The network consisted of two variable vacuum capacitors, one mounted in parallel and the other in series. The capacitors enabled nearly optimal matching when the electrical conductivity of gaseous plasma was high, *i.e.*, in the H-mode. These conditions were achieved in the plasma reactor at an oxygen pressure of 33 Pa and a forward discharge power of over 500 W.

### 2.3. Plasma Characterization

The plasma reactor was equipped with a residual gas analyzer (RGA). Mass spectra were measured with a PrismaPlus QMG 220 Residual Gas Analyzer from Pfeiffer Vacuum (Asslar, Germany). The analyzer was mounted on a stainless steel chamber which was differentially pumped with a Hi-CubeTM Turbo pumping station (Pfeiffer Vacuum, Asslar, Germany) using a nominal pumping speed of 685 L/s. The RGA chamber was connected to the plasma reactor through a glass-tube flow restrictor (a capillary), which kept the pressure in the RGA chamber below 5 × 10^−4^ Pa. The pressure in the plasma reactor was 10 Pa. Continuous mass spectra taken during sample treatment were recorded in the 0 to 50 Atomic Mass Unit (AMU) range at a speed of 200 ms/AMU. The complete spectrum up to 50 AMU was therefore acquired in 10 s.

The light emitted by the plasma during sample treatment was monitored by optical emission spectroscopy (OES). An AvaSpec-3648 fiber optic spectrometer from Avantes (Apeldoorn, The Netherlands), based on AvaBench-75 symmetrical Czerny-Turner design with 3648 pixel CCD detector array, was used. Its resolution was 0.5 nm over a wavelength range of 200 to 1100 nm. The integration time was set to 1 s when the plasma was in E-mode (weak light emission) and 1 ms when the plasma was in H-mode (much stronger emission). The OES spectrometer was connected through an optical fiber to the collimating lens, which was positioned approximately 1 cm from the plasma reactor, directly above the samples and perpendicular to the sample surface. The OES spectra were recorded every 2 s during the plasma treatment in E-mode. The spectra were not calibrated for the spectral sensitivity of the spectrometer.

The density of charged particles and neutral oxygen atoms in the ground state was measured in an empty chamber filled with oxygen using a home-made double electrical probe and a laser-heated catalytic probe supplied by Plasmadis Ltd. (Ljubljana, Slovenia). The probes operated only for the E-mode of the gaseous discharge because the thermal load in the H-mode was too high to measure plasma parameters.

### 2.4. Surface Characterization

Samples were characterized by XPS, water contact angle measurements and measurements of the water drop absorption time.

We used a TFA XPS from Physical Electronics (München, Germany). The samples were excited with monochromatic Al K_α1,2_ radiation at 1486.6 eV over an area of 400 µm^2^. Photoelectrons were detected with a hemispherical analyzer positioned at a 45° angle with respect to the normal of the sample surface. XPS survey spectra were measured at a pass-energy of 187 eV using an energy step of 0.4 eV, and high-resolution spectra were measured at a pass-energy of 23.5 eV using an energy step of 0.1 eV. An additional electron gun was used for surface neutralization during XPS measurements. All spectra were referenced to the main C1s peak. The measured spectra were analyzed using MultiPak v8.1c software (Ulvac-Phi Inc., Kanagawa, Japan, 2006) from Physical Electronics, which was supplied with the spectrometer.

The surface wettability was measured immediately after plasma treatment by determining the water contact angle (WCA) with a demineralized water droplet with a volume of 2 μL. Contact angles were measured with a See System goniometer (Advex Instruments, Brno, Czech Republic). For each sample, five measurements were taken to minimize the statistical error. The contact angles were determined by the software supplied by the producer. The absorption time was measured by high speed acquisition of images with a capture rate of 10 frames per second.

## 3. Results and Discussion

**Figure 4:**
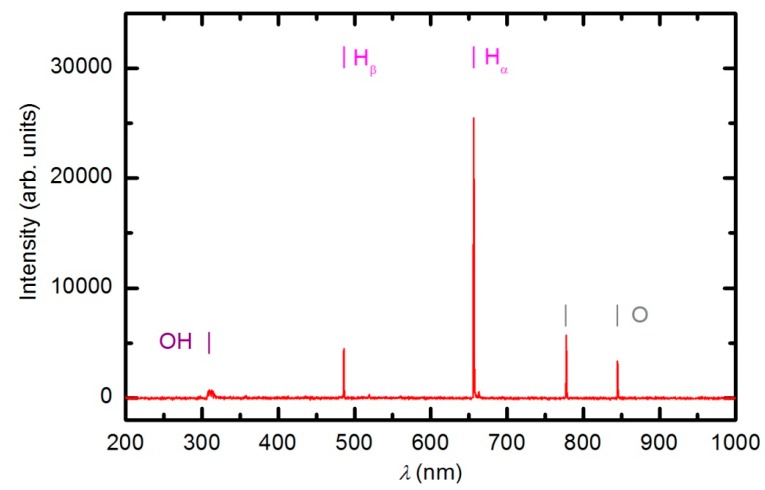
An optical spectrum of plasma created in the empty chamber at a forward power of 150 W and a pressure of 33 Pa.

The plasma in E-mode was characterized using optical emission spectroscopy, a catalytic probe and an electrical probe prior to experiments with polymer samples. Optical spectroscopy is a qualitative technique, whereas the probes give quantitative results. An optical spectrum of the plasma created at 33 Pa and a forward power of 150 W without a sample is shown in Figure 4.

The spectrum reveals extensive emission arising from neutral oxygen and hydrogen atoms as well as a weak band in the UV region at 309 nm. This band corresponds to radiation from the excited OH radicals formed in the plasma as a result of the dissociation of water molecules caused by electron impact. The absence of nitrogen bands confirms that there is negligible leakage of the plasma reactor. The origin of the water is a slow desorption from internal reactor surfaces. The experimental setup does not allow for baking, therefore, the residual atmosphere in both the plasma and RGA chambers is predominantly water vapor. The concentration of water in the plasma reactor cannot be determined by mass spectrometry because the water molecules entering the RGA originate from both the plasma and RGA chambers. The water partial pressure can only be estimated by considering the base pressure in the plasma chamber, which is approximately 1 Pa. The partial pressure of water is therefore much smaller than that of oxygen. The optical spectrum presented in Figure 4 indicates the high dissociation of both H_2_O and O_2_ molecules under plasma conditions. At a pressure of 33 Pa and forward power of 150 W, the absolute value of the O-atom density was determined with the catalytic probe to be approximately 1.2 × 10^21^ m^−3^. Such a high density is explained by extensive dissociation upon electron impact and the poor association of O atoms due to heterogeneous surface recombination. The association in the gas phase is negligible at 33 Pa because the reaction requires a three-body collision to preserve energy and momentum [21,34]. A forward power of 150 W does not allow for a high ionization fraction because the RF generator is mismatched. The reflected power meter on the generator showed a reflected power of 115 W; thus, the real discharge power was only approximately 35 W. The density of charged particles at these conditions was estimated using the double electrical probe to be only 4 × 10^16^ m^−3^.

PURPEG polymer samples were placed in the middle of the plasma reactor, directly under the collimating lens used for OES. The chamber was pumped down to a pressure of approximately 1 Pa and then filled with commercially available oxygen. The pressure was set to 33 Pa. Samples were treated with plasma generated with various forward RF power levels.

**Figure 5:**
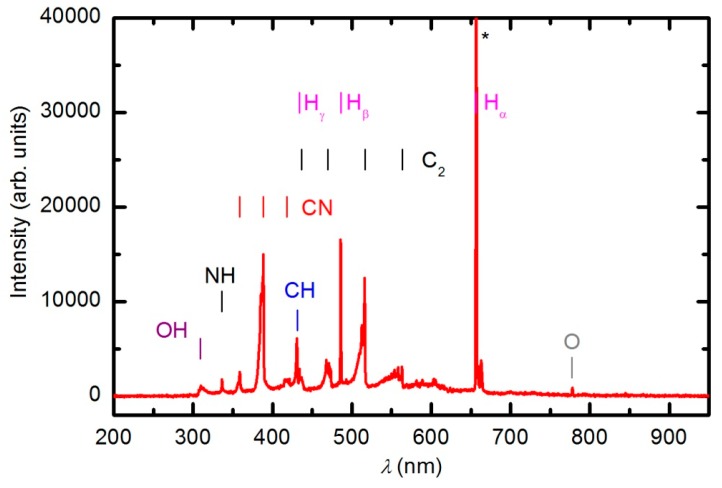
OES spectrum of the plasma during the etching of a PURPEG sample with oxygen plasma generated at 900 W forward RF power.

We attempted to treat samples in the powerful plasma created in H-mode at a forward power of approximately 900 W. The RF generator was matched well, and the reflected power was only 85 W. Such a powerful discharge created plasma of high luminosity. The integration time of the optical spectrometer was only 1 ms. Figure 5 shows the optical spectrum acquired through treatment of the polymer sample at 900 W. The absence of oxygen atomic lines indicate a rather low electron temperature, which is typical for RF plasma in H-mode. The radiation from O atoms arises from excited states of excitation energy approximately 14 eV, thus it is highly unlikely that electrons of low temperature excite the radiative states. The optical emission was much larger for other plasma particles. Apart from the hydrogen Balmer series (H_α_ was left to saturation), there were emissions from CH, CN, OH and NH radicals as well as C_2_ dimers. The presence of the dimers, CN radicals, and NH radicals indicates the thermal degradation of the polymer sample. If samples were oxidized, the predominant radiation would be from CO radicals. No mass spectrum was recorded during the experiment in H-mode because the sample visually degraded before the spectrum could be acquired. Therefore, sample treatment in H-mode at a real power close to 800 W caused thermal degradation rather than functionalization of the polymer sample. A similar thermal degradation of the PURPEG samples was also observed in H-mode ammonia plasma [33].

**Figure 6:**
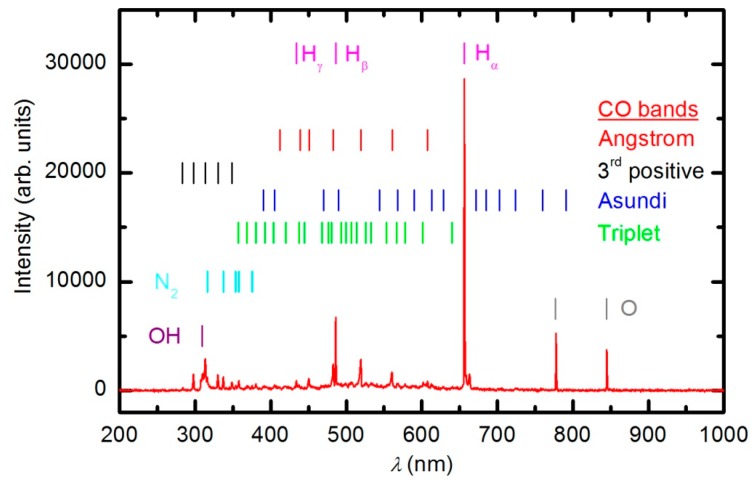
OES spectrum after 30 s of plasma treatment at 33 Pa and forward power of 150 W.

**Figure 7:**
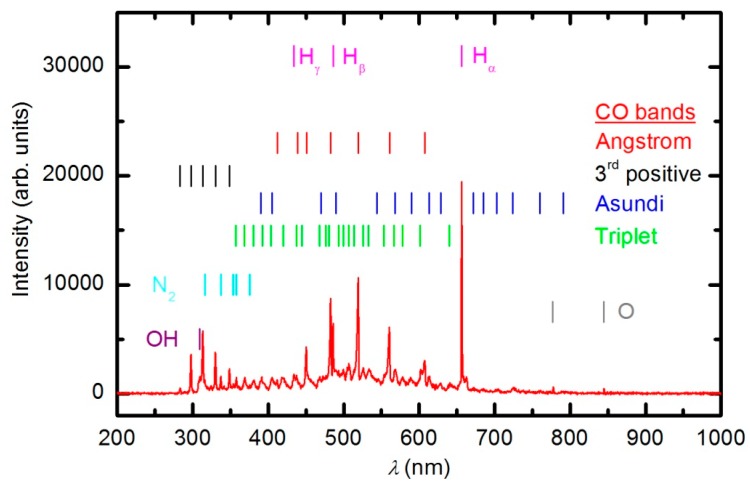
OES spectrum after 600 s of plasma treatment at 33 Pa and forward power of 150 W.

Numerous samples were treated with oxygen plasma in E-mode. Typical OES spectra of oxygen plasma during the sample treatment in E-mode at a forward power of 150 W are shown in Figure 6 and Figure 7. The optical spectrum just after turning on the discharge was identical to the spectrum acquired in an empty chamber (Figure 4). The spectrum in Figure 4 comprises only a few spectral features: hydrogen Balmer lines, an OH band (A^2^Σ–X^2^Π transition) at 309 nm and oxygen atom lines at 777.4 and 844.6 nm corresponding to transitions 3p5P–3s5S° and 3p3P–3s3S°, respectively. The presence of oxygen emission lines is obvious because the chamber was filled with oxygen. However, the OH band and Balmer lines are observed because of the water vapor in the system, which is present due to the water desorption from reactor walls and the sample.

As the plasma treatment continued, some new spectral features emerged. Figure 6 reveals the spectrum after 30 s of plasma treatment at a forward power of 150 W. Between 300 and 400 nm, a barely visible N_2_ second positive band (C^3^Π_u_–B^3^Π_g_) indicates that the sample also contains nitrogen. All other subsequent spectral features are attributed to CO radicals, which can be explained by etching of the sample [35,36]. In the UV region of the spectra, there is a 3^rd^ positive band, which belongs to the transitions between b^3^Σ^+^ and a^3^Π states. The central part of the spectrum from 350 to 800 nm is dominated by a broad continuum, which is known to be composed of overlapped bands of three CO systems: Angstrom (B^1^Σ^+^–A^1^Π), Triplet (d^3^Δ–a^3^Π) and Asundi (a’^3^Σ^+^–a^3^Π) bands [37,38]. The intensity of the CO bands in Figure 6 is a rather weak, qualitative indication that the amount of reaction products (CO_2_ and CO) is small in the initial stage of polymer processing.

**Figure 8:**
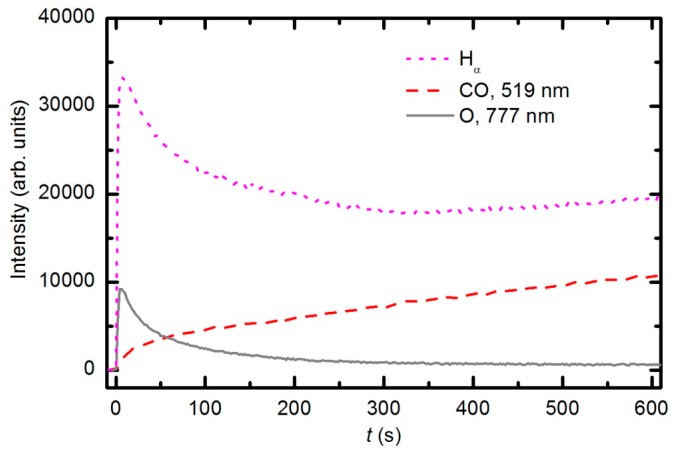
Time evolution of the CO emission peak (519.5 nm), O (777.4 nm) line and Balmer Hα line during sample etching with oxygen plasma generated at a pressure of 33 Pa and 150 W forward RF power.

Figure 7 represents the OES spectrum of plasma after a prolonged treatment of 600 s. The spectrum is similar to the one acquired at 30 s; however, the intensity of CO bands compared to O lines is much stronger. This observation is explained by the consumption of oxygen and accumulation of reaction products in the plasma chamber. Optical spectra were acquired continuously during sample treatment, and the intensity of the major features *versus* treatment time is presented in Figure 8. The intensity of the oxygen line at 777 nm and the H_α_ signal decreased with treatment time for the first few minutes and then stabilized, whereas the intensity of the CO bandhead at 519.5 nm (CO Angstrom line (0,2)) continued to increase. The behavior of the selected lines presented in Figure 8 indicates the etching of the polymer during plasma treatment. However, no firm conclusions about the gas composition can be drawn because the technique is qualitative. Furthermore, the intensity of emission lines depends on the electron temperature, which in turn depends on the gas composition. Unfortunately, the home-made double electrical probe does not allow for reliable determination of the electron temperature.

**Figure 9:**
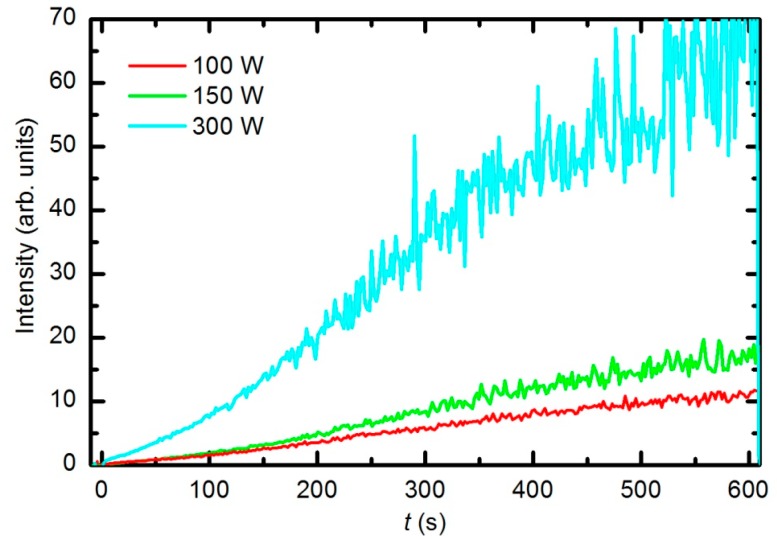
Time evolution of the CO emission peak (519.5 nm) normalized by the O (777.4 nm) line during sample treatment with oxygen plasma; the RF forward power is shown in the key.

The optical spectra were also recorded at different forward power levels of 100, 150 and 300 W. Considering the measured reflected power levels, these values correspond to real power levels of 25, 35 and 70 W, respectively. Figure 9 presents the time evolution of the CO Angstrom line (0,2 vibrational transitions) normalized by the time evolution of the oxygen atom line (777.4 nm) for each RF power level. We conclude that the etching rate is larger when the forward RF power is higher. At higher power levels, the densities of both neutral atoms and ions are larger [39], which explains the observed result.

**Figure 10:**
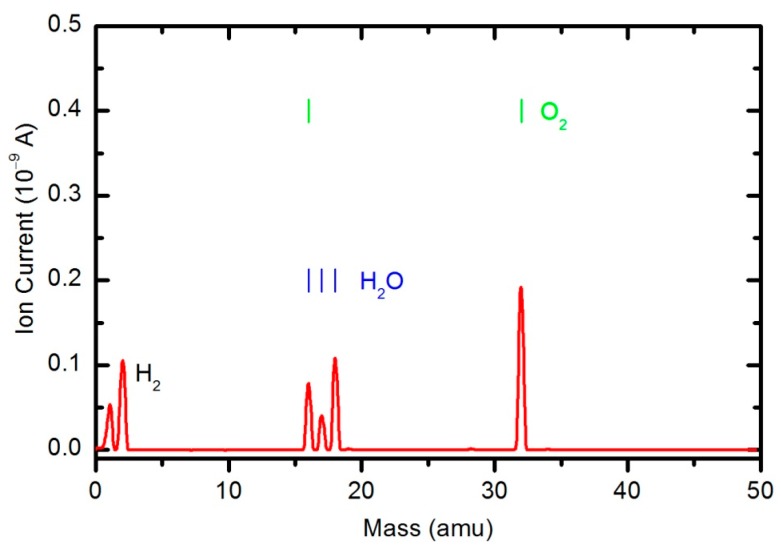
A mass spectrum measured in an empty chamber filled with oxygen at pressure 33 Pa and forward power of 150 W.

**Figure 11:**
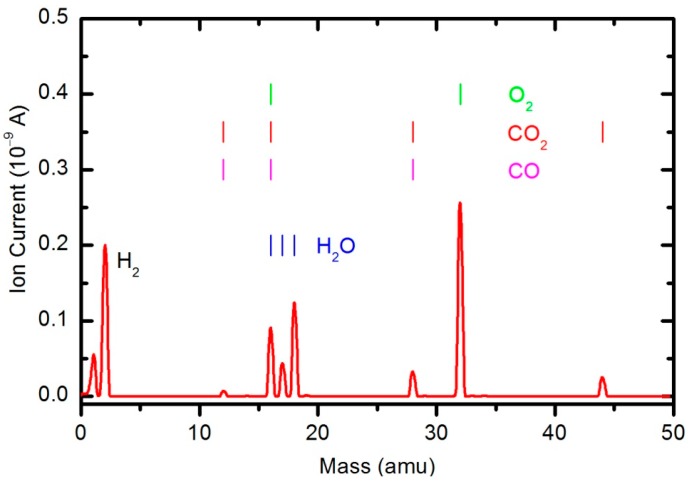
A mass spectrum measured in a chamber loaded with PURPEG samples after plasma treatment for 30 s. The pressure was 33 Pa and forward power 150 W.

**Figure 12:**
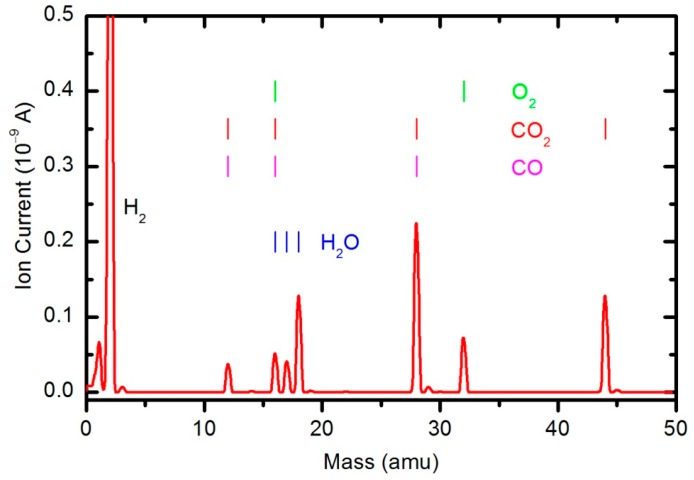
A mass spectrum measured in a chamber loaded with PURPEG samples after plasma treatment for 600 s. The pressure was 33 Pa and forward power 150 W.

The results of the OES measurements as summarized in Figure 8 and Figure 9 do not reveal the absolute concentration of stable gaseous molecules in the plasma chamber during polymer sample treatment. To quantify the results, we also performed measurements by mass spectrometry. Figure 10 shows a mass spectrum that was measured in an empty chamber filled with oxygen at a pressure of 33 Pa, and the spectra shown in Figure 11 and Figure 12 were obtained in a chamber containing samples after 30 and 600 s of plasma treatment, respectively. The forward RF power was 150 W.

**Figure 13:**
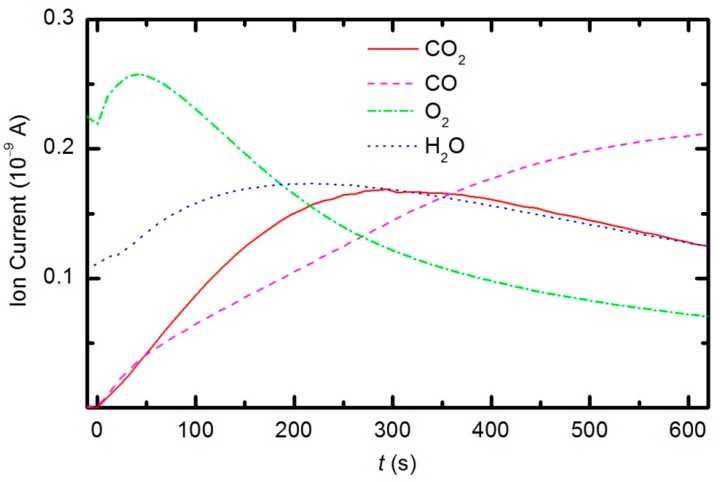
Time evolution of CO_2_, CO, O_2_ and H_2_O partial pressures during samples treatment with oxygen plasma at 33 Pa and 150 W forward RF power.

Peaks attributed to H_2_, H_2_O, O_2_, CO_2_ and CO can be observed in the mass spectra. The RGA spectrum recorded in an empty chamber contains only H_2_, H_2_O and O_2_ peaks. Because of the cracking pattern of the RGA, molecules are not detected as only one mass peak. All of the detected peaks for a certain molecule are marked in Figure 10, Figure 11 and Figure 12. For example, the peak at mass 16 is the sum of the contributions from the following stable molecules: H_2_O, O_2_, CO_2_ and CO. Fortunately, the molecules H_2_O, O_2_ and CO_2_ can be monitored by directly observing masses 18, 32 and 44, respectively. To observe CO molecules, which are produced during the plasma treatment of a sample, one must subtract 11.4% of the mass 44 peak intensity from mass 28. Thus, it is possible to quantify the evolution of the molecules’ partial pressures in the discharge chamber. The result is presented in Figure 13. The results indicate behavior that is consistent with the observations from OES (Figure 8). The O_2_ mass peak decreases with time because oxygen is consumed during the oxidation of the sample. The products of sample etching are CO and CO_2_, whose concentration increases with time. Although CO_2_ reaches a maximum at approximately 250 s and then starts to decrease, CO continues to increase, thus indicating etching of the sample.

**Figure 14:**
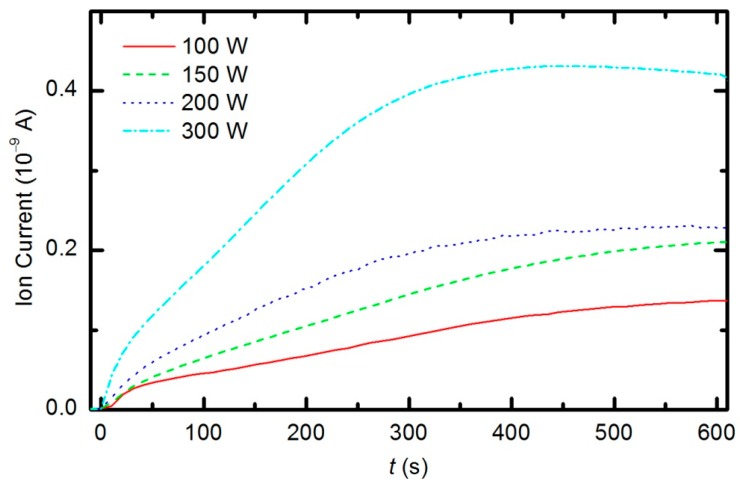
Time evolution of CO during sample treatment with oxygen plasma generated at various forward RF power levels.

**Figure 15:**
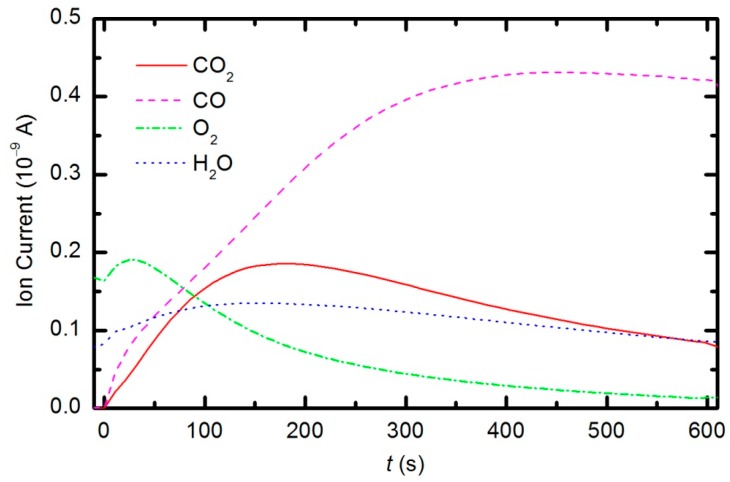
Time evolution of CO_2_, CO, O_2_ and H_2_O partial pressures during sample treatment with oxygen plasma at 33 Pa and 300 W forward RF power.

The etching of the polymer by plasma was also monitored by following the time evolution of the CO concentration. Figure 14 represents the partial pressure of the CO molecule *versus* treatment time at the power levels 100, 150, 200 and 300 W. The etching rate is higher if the sample is treated with plasma that is generated with a higher forward RF power. At 300 W, the concentration of CO reaches a maximum after a certain treatment time (approx. 350 s) and remains nearly constant with prolonged treatment. A feasible explanation for this observation is that all of the oxygen inside the discharge chamber has been consumed for polymer oxidation; therefore, CO can no longer be produced. After consuming the oxygen, the CO concentration slowly decreases as it is pumped out through the RGA chamber. This conclusion is supported by the time evolution of partial pressures at 300 W (Figure 15). One can observe practically no remaining oxygen after approximately 350 s of treatment. Approximately 5% of the initial partial pressure of oxygen can easily be explained by the dissociation of CO in the discharge and recombination of O-atoms into O_2_ on the surface of the discharge chamber.

**Figure 16:**
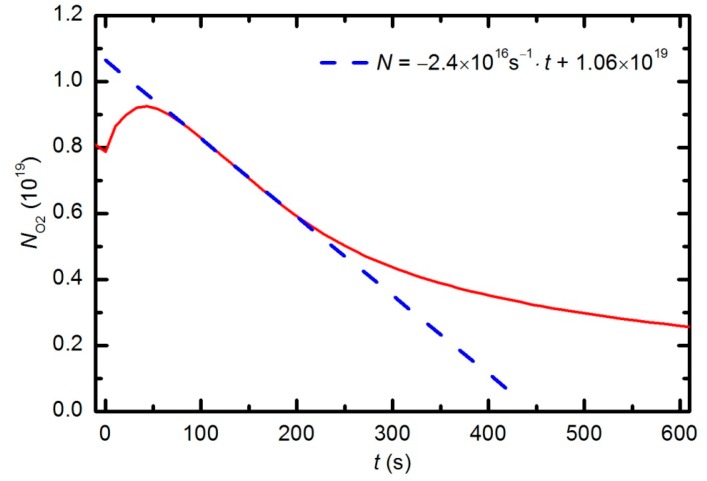
The slope of the oxygen partial pressure during the first few minutes of plasma treatment at 33 Pa and 150 W.

The behavior of the oxygen partial pressure in Figure 13 is rather unexpected and thus worth discussing. The oxygen partial pressure first increases with time, reaches a maximum after approximately 50 s of plasma treatment, and then decreases with further treatment. The increase in O_2_ partial pressure during the first 50 s is probably an artifact of thermal effects. The discharge power initially heats the gas inside the plasma chamber. The real power (35 W) dissipated in the plasma reactor causes slow heating of the gas until such a temperature is achieved that heating is balanced by cooling with ambient room-temperature gas. The pressure in the plasma chamber depends on the density of molecules and gas temperature: *p* = *nk*_B_*T*. The oxygen partial pressure in Figure 13 increased by approximately 10% from the initial value over a period of 50 s; therefore, one could estimate the gas temperature to be approximately 330 K at that treatment time. This gas temperature is an underestimate because oxygen is continuously being lost due to chemical reactions with the polymer sample (etching). A better estimate would account for this effect. The simplest method is to extrapolate the time derivative of the oxygen partial pressure towards zero, as shown in Figure 16. The difference in the measured oxygen partial pressure and the cross-section of the extrapolation line with the y-axis is approximately 30%; thus, a better estimate of the gas temperature after a 50 s treatment time is 390 K. This value is typical for cold, non-equilibrium gas plasma.

Figure 16 can also be used to estimate the etching rate. As a rough approximation, one could assume that each oxygen molecule that is lost through polymer oxidation captures 3 polymer atoms (reaction –CH_2_– + O_2_→CO + H_2_O). The true polymer composition is much more complex; therefore, the assumption is an oversimplification and underestimates the etching rate. However, it does allow us to make a rough estimate of the etching rate. The rate is calculated as follows:

(1) $$\zeta =3\frac{dN_{O_{2}}}{dt}\cdot \frac{x_{\text{mon}}}{S\cdot N_{\text{mon}}}$$

where d*N_O_*_2_/d*t* is the slope of the line in Figure 15, *x*_mon_ is the thickness of an atomic monolayer in the polymer, *S* is the surface area of the sample and *N*_mon_ is the surface density of atoms in the solid material. Considering the measured value (d*N*_O2_/d*t* = 2.4 × 10^16^ s^−1^ in Figure 15) of *S* = 2 × 10^−4^ m^2^, and typical values for *x*_mon_ = 3 × 10^−10^ m and *N*_mon_ = 1 × 10^19^ m^−2^, the etching rate is estimated to be 10 nm/s. This etching rate is typical for polymers and has been measured by much more sophisticated techniques, such as quartz crystal microbalance [40].

It might be possible to estimate the etching rate in a simplified manner, *i.e.*, by weighing a sample before and after plasma treatment. This procedure is common for smooth, inorganic materials that do not adsorb significant amounts of water. Our samples, however, are highly porous polymers; therefore, they adsorb significant amounts of water. The water molecules slowly desorb from the sample due to both vacuum conditions (equilibrium partial pressure of water vapor at 300 K is over 3000 Pa) as well as heating of the sample.

The behavior of the curve in Figure 15 indicates linearity up to a treatment time of approximately 220 s (given a correction for thermal effects, see explanation above) and then a lower absolute value of the time derivative. The curve reflects the etching kinetics: up to approximately 220 s, the etching rate *ζ* does not depend on time, and thereafter, it slowly decreases with increasing treatment time. The deviation from linearity is explained by a shortage of reactive oxygen species, which is due to the consumption of O_2_ molecules and the formation of more stable CO_2_ and CO molecules. The etching rate is therefore initially limited by the intensity of the surface reactions, and after approximately 220 s, by the arrival of reactive species onto the polymer surface.

The major reactants in the plasma created by these particular conditions are neutral oxygen atoms. They abound in the plasma at a density on the order of 10^21^ m^−3^. The corresponding flux of O-atoms on the surface is over 10^23^ m^−2^·s^−1^; therefore, they should be capable of removing thousands of monolayers in a second. The positively charged ions are minor reactants, but they may contribute to etching acceleration due to synergistic effects with atomic oxygen and UV radiation from the plasma. The theoretical limit of the etching rate by O_2_^+^ ions can be estimated from the measured density of charged particles. Because the Debye length in our plasma is orders of magnitude smaller than the lateral dimension of our samples, an approximation of infinitely large samples can be adopted. The ion flux onto the surface in this approximation is *j* = ¼ *n*_+_<*v*_+_>, where *n*_+_ is the ion density in plasma and <*v*_+_> is the ions’ average random velocity (almost 450 m/s). If all ions react chemically with the polymer, the etching rate would be:

(2) $$\zeta_{+}=3j\frac{x_{mon}}{N_{mon}}=0.4\;\text{nm}/s.$$

The measured value is an order of magnitude larger; therefore, it is clear that the ions alone are not responsible for all of the polymer etching. The measured etching rate is thus explained by the interaction of PURPEG polymer with neutral reactive species (*i.e.*, O-atoms in the ground state) and perhaps by synergistic effects with ions and/or UV radiation.

**Figure 17:**
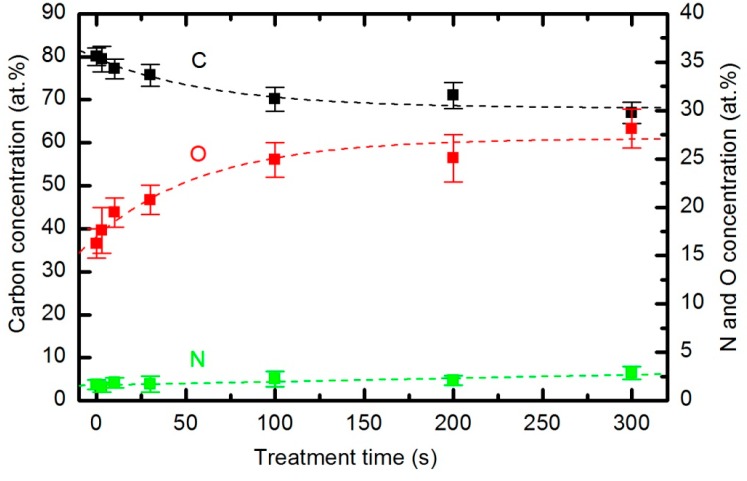
The composition of the surface film as calculated from the XPS survey spectra. The pressure was 33 Pa and the forward power was 150 W.

Whatever the mechanism involved, the oxygen plasma treatment not only causes etching of the polymer but also results in functionalization [41,42] that modifies the wettability of the material. The functionalization in our case was investigated by XPS. Survey spectra were measured for each sample soon after plasma treatment to minimize the influence of any ageing effects (hydrophobic recovery). All characterized samples were treated at a pressure of 33 Pa and a forward power of 150 W. The composition of the surface film as calculated from the XPS survey spectra is shown in Figure 17. The results are not impressive. The original concentration of carbon is approximately 80 atomic %. It decreases monotonously, and the concentration after a prolonged treatment of 5 minutes is 65 atomic %. The opposite behavior is observed for oxygen and nitrogen. The nitrogen concentration is only up to 2 atomic %; therefore, considering the accuracy of this technique, the relative variations are not good representations of surface changes. Consequently, we limit our discussion to the evolution of the oxygen concentration. The oxygen concentration is approximately 17 atomic % for untreated samples, increasing to approximately 23 atomic % in the first 10 s of plasma treatment. The concentration continues increasing slowly until it reaches approximately 27 atomic % for the sample treated for 300 s. The survey spectra were measured at 3 spots on each sample, and the error bars in Figure 17 indicate the statistical error only. The results presented in Figure 17 therefore indicate that functionalization is not the predominant mechanism of interaction between reactive species from the oxygen plasma and the polymer surface. This type of polymer PURPEG does not exhibit much tendency to functionalize, which is explained by the instability of the oxidized surface material, which tends to desorb from the surface rather than accumulate much oxygen.

**Figure 18:**
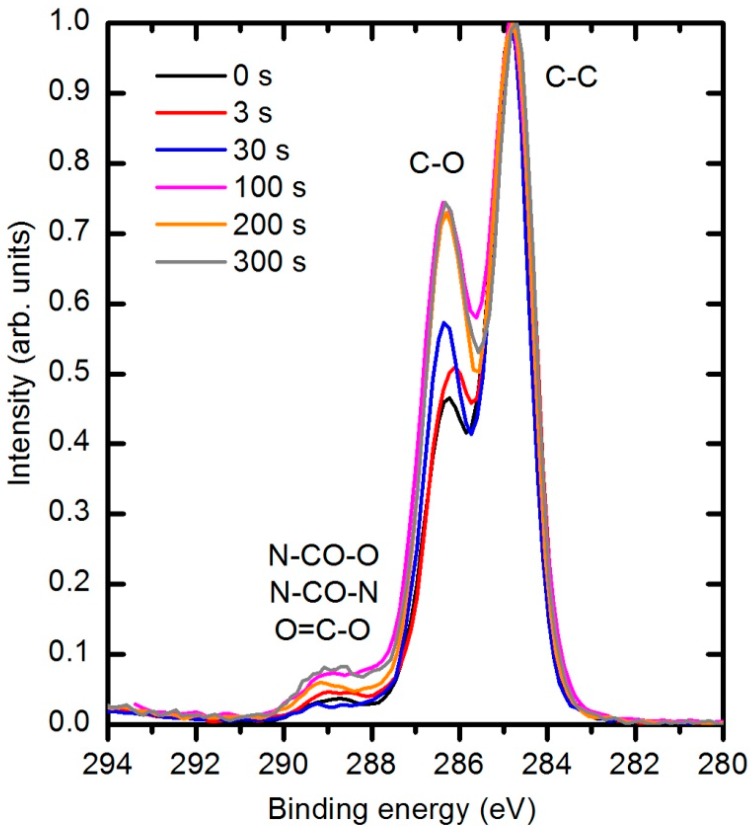
Evolution of XPS C1s spectra for PURPEG samples as a result of treatment with oxygen plasma at 33 Pa and 150 W.

The types of newly formed functional groups can be deduced from XPS high resolution C1s peaks. Figure 18 represents the evolution of this peak for different plasma treatment times. Three sub-peaks are observed. The largest peak at 284.8 eV corresponds to bonds between carbon atoms, the second peak at 286.4 eV to the single bonds of either C–N or C–O, and the small peak at 289 eV to –NH–CO–O– and –NH–CO–NH– groups in the original polymer structure. Because the nitrogen concentration is small and does not change with plasma treatment, it is clear that the modifications in the C1s peak are due to interactions with oxygen. Both peaks at 286.4 and 289 eV increase with plasma treatment time. There is no reason for the increased concentration of the peak at 289 eV; therefore, one can conclude that the increased peak at this binding energy corresponds to the formation of a O=C–O carboxylic or ester group. Unfortunately, the technique does not allow us to distinguish between these groups because the difference in binding energy between them is too small [43,44,45]. The increasing intensity of the peak at 286.4 eV indicates that hydroxyl groups are also formed during plasma treatment.

**Figure 19:**
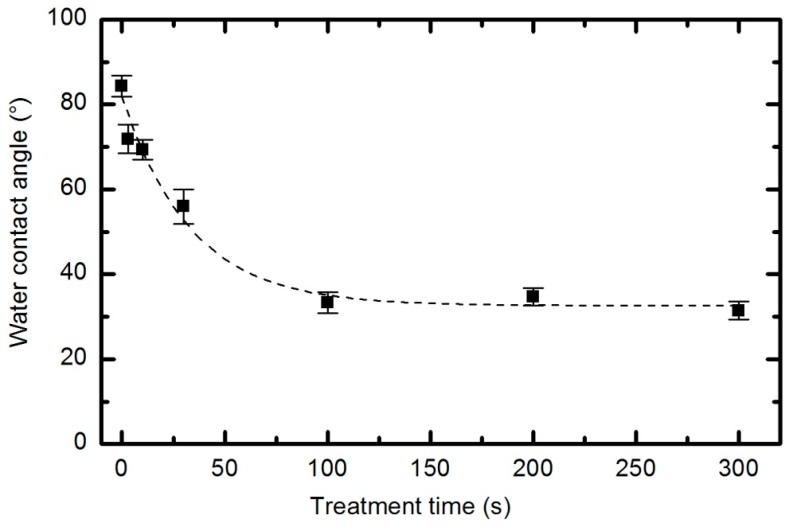
Water contact angle *versus* plasma treatment time at 33 Pa and 150 W.

**Figure 20:**
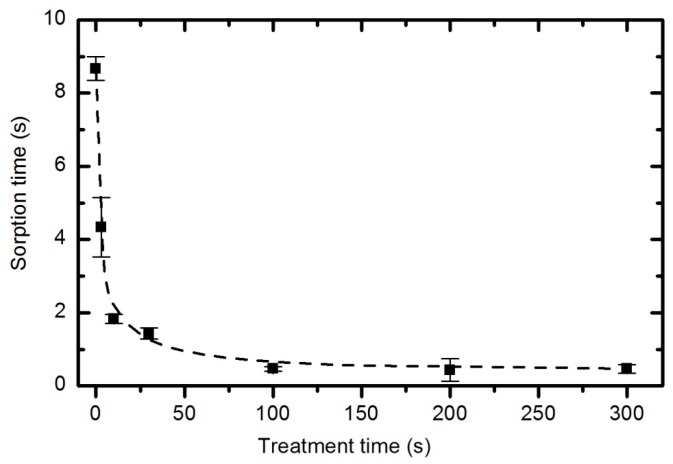
Absorption time of a water droplet *versus* plasma treatment time at 33 Pa and 150 W.

Irrespective of the functional groups formed and despite the fact that functionalization is not reasonable, the plasma treatment caused significant modifications to the surface energy and the sorption kinetics. The water contact angle *versus* plasma treatment time for the case of an oxygen pressure of 33 Pa and a forward power of 150 W is plotted in Figure 19, with the corresponding water droplet absorption time in Figure 20. The error bars in both figures indicate statistical error resulting from 5 repetitions.

The water contact angle (Figure 19) roughly follows the concentration of oxygen on the film surface (Figure 17). The contact angle for the untreated sample is approximately 85°, indicating the moderately hydrophobic character of as-synthesized PURPEG. The water contact angle drops to approximately 75° even with only 3 s of plasma treatment. Such a rapid effect is explained by a huge flux of O-atoms onto the sample surface. The segments of the polymer surface which were directly exposed to oxygen plasma quickly chemisorbed oxygen atoms and became saturated with polar functional groups. Further exposure causes etching, as revealed in Figure 5, Figure 6, Figure 7, Figure 8, Figure 9, Figure 10, Figure 11, Figure 12, Figure 13, Figure 14 and Figure 15. As the treatment proceeds, the saturation of the polymer surface with oxygen functional groups slowly propagates into the pores. Once the entire surface in contact with the water droplet saturates, the contact angle assumes a constant value of approximately 30°, which is typical for this type of material. This value is much higher than for most aromatic polymers and comparable to the values for aliphatic polymers. Specifically, polymers with a high concentration of aromatic rings, such as polyethylene terephthalate or polystyrene, usually exhibit contact angles of approximately 10° when smooth and much smaller when nanostructured [46,47,48]. PURPEG does not contain aromatic rings but just normal cyclohexane rings, which could explain the rather poor activation of the surface as revealed in Figure 19. From application point of view, such a poor wettability may be beneficial because the best cell proliferation has been observed for moderately hydrophilic polymers [10,11]. Furthermore, a rather slow decrease in the contact angle with respect to treatment time allows us to select the desired wettability over a broad range of contact angles between 70° and 30°, corresponding to treatment times of 10 to 100 s.

Improved sorption of water droplets does not require the saturation of the entire material with functional groups. Figure 20 reveals that the water droplet absorption time quickly drops from almost 9 s (for the untreated material) to 2 s (for samples exposed to plasma for 10 s). Further treatment does not have a significant influence on the adsorption time because even after 5 minutes of plasma treatment, the adsorption time remains at approximately 0.5 s.

## 4. Conclusions

The hydrophilization of a polyurea copolymer foam using oxygen plasma treatment was investigated. Treatment with plasma, when created by a powerful inductively coupled RF discharge in the H-mode, caused rapid degradation of samples due to extensive heating. Weaker plasma performed much better. At a discharge power of 35 W, the functionalization of porous samples progressed rather slowly, as the water contact angle approached a constant value of approximately 30° in one minute of plasma treatment. The soaking kinetics, however, were significantly improved even after a few seconds of plasma treatment. The interaction between plasma and polymer material was deduced from measured plasma parameters. Weak etching was observed simultaneously with functionalization. The etching led to a depletion of oxygen at the expense of carbon oxides. Initially, carbon dioxide was the predominant species formed; however, prolonged treatment caused incomplete oxidation because carbon monoxide was the dominant species after longer treatment times. The etching intensity was first limited by surface reactions, but for prolonged treatment, the limitations were due to the flux of reactive oxygen species onto the sample surface. In the initial treatment stages, the etching rate did not depend on treatment time; however, as the flux of oxygen species stabilized, the etching rate decreased with treatment time. The results clearly show that the treatment is suitable for tailoring surface properties in a controlled manner. The required surface wettability of a sample can be obtained with the proper selection of treatment time.

## Abbreviations

The following abbreviations are used in this manuscript:

AMU	Atomic Mass Unit
OES	Optical Emission Spectroscopy
PCL	Polycaprolactone
PGA	Polyglycolic Acid
PLA	Polylactic Acid
PURPEG	Polyurethane/urea copolymers based on poly(ethylene glycol)
RGA	Residual Gas Analyzer
RF	Radio Frequency
XPS	X-ray Photoelectron Spectroscopy
